# In Vitro Evaluation of the Performance of Self-Adhesive Resin Cements on Zirconia

**DOI:** 10.3390/jfb17020070

**Published:** 2026-01-29

**Authors:** Jiyoung Kwon, Hosung Lee, Hyun-Jung Kim, Kyoung-Kyu Choi

**Affiliations:** 1 Department of Conservative Dentistry, Kyung Hee Dental Hospital, Seoul 02447, Republic of Korea; jykt55@naver.com; 2 Department of Conservative Dentistry, Armed Forces Hongcheon Hospital, Hongcheon 25164, Republic of Korea; 46876@naver.com; 3 Department of Conservative Dentistry, School of Dentistry, Kyung Hee University, Seoul 02447, Republic of Korea

**Keywords:** self-adhesive resin cement, zirconia, 10-methacryloyloxydecyl dihydrogen phosphate, primer, bond strength

## Abstract

This study evaluated the bond strength of self-adhesive resin cement (SARC) containing 10-methacryloyloxydecyl dihydrogen phosphate (MDP) and calcium silicate, with and without zirconia primer, before and after thermocycling. Sintered zirconia specimens (*n* = 180) were sequentially polished, sandblasted, and bonded with TheraCem (TC), Clearfil SA Luting (SA), or Rely X U200 (RU), with and without Z-Prime Plus primer. Specimens were stored in water at 37 °C or subjected to 10,000 thermocycles (5–55 °C). Shear bond strength (SBS), failure modes, fracture surfaces, flexural strength, and Vickers hardness were assessed. Bonding performance was governed by material-specific interactions rather than a complex three-factor interplay between resin cement type, primer application, and thermocycling. SBS followed the order TC > SA > RU and was significantly higher with primer application. Thermocycling significantly reduced SBS in all groups. Premature failure occurred in the RU and SA groups. Mixed failure was predominant across all conditions. The flexural strength and Vickers hardness were highest in the RU group, followed by the TC and SA groups, with RU maintaining significantly higher hardness even after thermocycling. Overall, SARCs containing MDP and calcium silicate demonstrated favorable bonding performance, which was further enhanced by zirconia primer application.

## 1. Introduction

Establishing a stable interface between the tooth structure and the internal surface of indirect restorations is crucial for long-term durability [1,2]. However, achieving proper adhesion in indirect restorations is challenging because they require a technically complex cementation process, necessitating thorough conditioning of both the tooth and the intaglio surface of the restoration before cement application [3]. To overcome this challenge, extensive efforts have been made to simplify the procedure. The development of self-adhesive resin cements (SARCs) has eliminated the need for separate adhesive and etching steps, thereby reducing application time and improving clinical efficiency. These cements contain acidic functional monomers that facilitate bonding to the tooth surface while minimizing sensitivity during the cementation process [4].

Yttria-stabilized tetragonal zirconia polycrystal (Y-TZP) is widely used in restorative dentistry because of its favorable aesthetics, high strength, and biocompatibility [5]. Even when sufficient crown length and convergence angle provide acceptable retention with traditional cements, zirconia crowns present unique bonding challenges compared with conventional silica-based ceramics. Zirconia cannot be etched with hydrofluoric acid at the temperatures, durations, and concentrations commonly used in clinical practice, making traditional etching methods ineffective [6,7]. Furthermore, zirconia lacks a glassy phase, preventing the formation of chemical bonds through silanization, which is essential for durable adhesion in silica-based ceramics [8]. Additionally, the chemically inert surface of zirconia presents challenges in establishing a durable bond, necessitating physical and chemical surface treatments, such as sandblasting or the application of primers to enhance retention [9,10]. Consequently, the inherent material characteristics of zirconia significantly complicate clinical bonding processes.

To enhance adhesion to zirconia, various functional monomers such as 4-methacryloyloxyethyl trimellitate anhydride, phenyl-P, glycerol phosphate dimethacrylate (GPDM), and 2-hydroxyethyl methacrylate phosphate have been used. Among these monomers, 10-methacryloyloxydecyl dihydrogen phosphate (MDP) is the most extensively utilized and well-studied [11,12]. MDP possesses a hydrophilic phosphate group that facilitates mild acidic decalcification of tooth substrates and enables chemical interactions with the zirconia surface [9]. Simultaneously, its hydrophobic alkyl chain helps maintain an optimal balance between hydrophilicity and hydrophobicity, promoting compatibility with both the resin matrix and zirconia surface. The terminal methacrylate group allows for efficient polymerization with the resin component, contributing to durable bond formation [13,14].

According to previous research [9], MDP adheres to zirconia through multiple interaction mechanisms, including hydrogen bonding between oxo- and hydroxyl groups on the zirconia surface, ionic interactions between phosphate groups of MDP and zirconia, and hydrogen bonding involving non-ionized P–OH groups with zirconia or neighboring phosphate moieties. While these interactions collectively contribute to zirconia bonding, they do not contribute equally to bond strength. Studies using TiO_2_ model surfaces have shown that bidentate and energetically favored monodentate binding modes of long-chain phosphates such as 10-MDP play a predominant role by promoting dense surface packing and the formation of a more ordered interfacial structure [15]. These molecular-level interactions underpin the effectiveness of MDP-containing primers and resin cements in achieving reliable initial bonding to zirconia in clinical applications.

Consistent with this mechanistic understanding, recent studies have reported that self-adhesive resin cements (SARCs) containing MDP exhibit higher initial bond strengths than those formulated without MDP [16,17,18]. However, despite this favorable chemical affinity, the bond strength of both MDP-containing and non-MDP-containing SARCs to zirconia has been shown to decrease significantly after thermocycling [19,20,21]. This reduction suggests that incorporation of MDP alone may be insufficient to maintain long-term bond durability under aging conditions.

In particular, 10-MDP is characterized by a long alkyl spacer chain, which contributes to a more hydrophobic molecular architecture compared with other phosphoric-acid monomers such as GPDM and dipentaerythritol pentaacrylate monophosphate. Rather than directly enhancing pH neutralization, which is a water-mediated acid–base process influenced primarily by ion-leachable components in self-adhesive resin cements, the longer hydrophobic spacer of 10-MDP may be more relevant to limiting water sorption and improving resistance to hydrolytic degradation over time [14,22,23].

It remains unclear whether the use of an additional primer improves the bond strength between MDP-containing SARCs and zirconia. Yue et al. [24] and Lim et al. [25] reported that applying an MDP-containing primer in combination with an MDP-containing SARC produced a statistically significant increase in shear bond strength (SBS) compared with no-primer controls. The authors attributed this improvement to the augmented availability of the dihydrogen functional groups supplied by the MDP monomer in both the primer and cement, which facilitated chemical bonding with zirconium oxide. Even when MDP is incorporated into SARC, pretreating the zirconia surface with an MDP-functional monomer is advisable [20,26,27,28,29].

Conversely, Afrasiabi et al. [30] found no statistically significant difference (*p* > 0.05) between resin cements with and without MDP primers. This suggests that discrepancies in experimental methodology, material composition, and surface treatment protocols may account for these conflicting results, underscoring the need for further systematic investigations. In practice, manufacturers typically recommend the use of MDP-containing SARCs without a separate priming step, a guideline supported by comparative studies of MDP-containing and non-MDP-containing SARCs in zirconia restorations [31,32]. Since the introduction of the first self-adhesive resin cement (e.g., Rely X Unicem, 3M, St. Paul, MN, USA), these materials have incorporated ion-leachable fillers, such as calcium hydroxide-containing phases, designed to partially neutralize acidity during setting. Accordingly, acid-neutralization should be considered a shared characteristic of self-adhesive resin cements rather than a feature unique to a single formulation [33]. More recently, calcium silicate-containing SARCs, such as TheraCem (BISCO, Schaumburg, IL, USA), have been reported to exhibit alkaline behavior and sustained calcium ion release [34]. Although the presence of calcium-containing deposits on dentin surfaces does not constitute direct evidence of dentin remineralization, calcium ion release may contribute to buffering the local acidic environment and promoting mineral precipitation under favorable conditions. These effects may indirectly enhance interfacial stability and sealing performance, potentially contributing to improved long-term bonding durability.

Numerous studies have demonstrated a clear correlation between the bond strength and clinical performance, highlighting the importance of simultaneously evaluating the adhesive durability and other key physical properties. Among these properties, flexural strength and surface hardness, which are commonly assessed using flexural strength and Vickers hardness tests, play pivotal roles in determining the long-term success of restorative materials.

In this study, the flexural strength and Vickers hardness were evaluated as supplementary mechanical parameters to account for potential material-dependent influences on the bond strength outcomes. Flexural strength reflects the resistance of the cement to bending forces and may affect its ability to withstand repetitive occlusal loading within a thin cement layer [35,36,37]. Vickers hardness indicates resistance to localized deformation and surface damage, which can indirectly influence the integrity of the cement layer under masticatory forces [38,39,40].

Therefore, this study aims to evaluate the bonding performance of SARC with respect to the application of zirconia primers. The SBS was assessed both immediately and following thermocycling, depending on the type of cement used and whether a primer was applied. The null hypotheses of this study were as follows: (1) The type of self-adhesive resin cement has no significant effect on the shear bond strength to zirconia. (2) The application of a zirconia primer has no significant effect on the shear bond strength of self-adhesive resin cements to zirconia. (3) Artificial aging by thermocycling has no significant effect on the shear bond strength of self-adhesive resin cements to zirconia.

## 2. Materials and Methods

### 2.1. Materials

This study used Mazic Zirconia blocks (Vericom, Chuncheon, Republic of Korea, A2 Shade). Three SARCs were tested, namely, Rely X U200 (3M ESPE, Neuss, Germany), TheraCem (BISCO Inc., Schaumburg, IL, USA), and Clearfil SA Luting (Kuraray Noritake Dental Inc., Okayama, Japan). Additionally, an MDP-containing zirconia primer (Z-Prime Plus; BISCO Inc., Schaumburg, IL, USA) was applied to specific groups, whereas others were tested without primer application. The compositions of the materials are listed in Table 1.

A total of 180 zirconia specimens were sectioned from the pre-sintered zirconia discs using a high-speed diamond saw (IsoMet 5000, Buehler, Lake Bluff, IL, USA). Each specimen was sectioned into a rectangular prism (10 mm wide, 10 mm long, and 15 mm high), followed by sequential polishing with silicon carbide papers of 320, 600, and 1000 grit. Sintering was performed at 1500 °C using a ceramic furnace (Austromat 674i; DEKEMA Dental-Keramiköfen GmbH, Freilassing, Germany) according to the manufacturer’s instructions. One surface was sandblasted with 50 μm aluminum oxide (Al_2_O_3_) powder at 0.25 MPa for 15 s, followed by ultrasonic cleaning in ethanol for 3 min, rinsing with distilled water, and air-drying.

**Figure 1:**
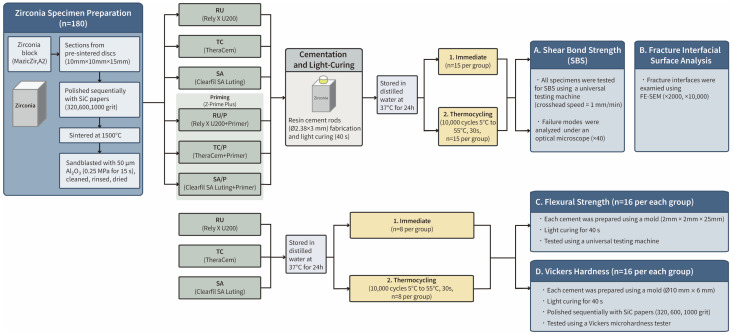
Experimental flow chart. TC, TheraCem; TC/P, TheraCem + primer; RU, Rely X U200; RU/P, Rely X U200 + primer; SA, Clearfil SA Luting; and SA/P, Clearfil SA Luting + primer; ⌀, Diameter; SBS, shear bond strength; SiC, silicon carbide.

The specimens were randomly assigned to six groups based on the type of SARC and the zirconia primer applied: RU (Rely X U200), RU/P (Rely X U200 + primer), TC (TheraCem), TC/P (TheraCem + primer), SA (Clearfil SA Luting), and SA/P (Clearfil SA Luting + primer). These groups are shown in Figure 1.

After cleaning, the zirconia primer was applied using a microbrush and air-dried. A resin cement rod (⌀2.38 mm × 3 mm) was fabricated on each specimen using a cylindrical Teflon mold within a bonding jig (Ultradent Products Inc., South Jordan, UT, USA). Light curing was performed using an LED curing unit (Elipar™ DeepCure-S; 3M) at an irradiance of 1300 mW/cm^2^ for 40 s. All specimens were stored in distilled water at 37 °C for 24 h and then divided into two subgroups (*n* = 15) based on artificial aging conditions: (1) immediate testing and (2) thermocycling (10,000 cycles between 5 °C and 55 °C; 30-s dwell time).

### 2.2. Shear Bond Strength

SBS was evaluated using a universal testing machine (AGS-X; Shimadzu Corp., Kyoto, Japan) at a crosshead speed of 1 mm/min. The load was applied to the interface with a knife edge until failure occurred in MPa (N/mm^2^). After testing, the specimens were examined under an optical microscope (SZN745 stereomicroscope; Sunny Optical Technology Co., Ltd., Shanghai, China) at 40× magnification to analyze the debonded surfaces. The failure modes were classified as adhesive (failure at the zirconia–resin cement interface) or mixed (adhesive failure combined with cohesive failure affecting >25% of the bonded surface). Specimens that failed prior to mechanical loading were classified as undergoing premature failure. These specimens were included in the SBS analysis and assigned an SBS value of 0 MPa, because they underwent complete interfacial failure before the application of stress. This approach was used to prevent overestimation of the bond strength and to reflect the weakest bonding performance in the statistical analysis. The premature failure rate was determined by calculating the percentage of specimens that failed before testing, relative to the total number of specimens in each group.

### 2.3. Fracture Interfacial Surface Analysis

The fracture interfaces of each group after the SBS test were examined using field-emission scanning electron microscopy (FE-SEM; S-4700, Hitachi High-Technologies Corp., Tokyo, Japan). All specimens were thoroughly dried and sputter-coated with platinum particles. SEM imaging was performed at magnifications of ×2000 and ×10,000 at an operating voltage of 10 keV to analyze the fracture modes.

### 2.4. Flexural Strength Test

Sixteen rectangular specimens were prepared for each resin cement using molds with dimensions of 25 mm × 2 mm × 2 mm (length × width × thickness). After storage in water at 37 °C for 24 h, eight specimens from each group were immediately tested, whereas the remaining eight underwent 10,000 thermocycles between 5 and 55 °C before testing. Flexural strength testing was conducted in accordance with the International Standard ISO 4049 for resin-based dental materials, using a three-point bending configuration and bar-shaped specimens [41]. A universal testing machine (AGS-X; Shimadzu Corp., Kyoto, Japan) was used for evaluation at a crosshead speed of 1 mm/min.

### 2.5. Vickers Hardness Test

For the Vickers hardness test, each resin cement was placed in a circular mold with dimensions of 10 mm × 6 mm. Prior to the testing, the specimen surfaces were sequentially polished with silicon carbide papers of 320, 600, and 1000 grit to obtain a flat and standardized surface. After immersion in water at 37 °C for 24 h, eight specimens from each group were immediately tested, whereas the remaining specimens were tested after 10,000 thermocycles between 5 and 55 °C. All specimens were evaluated using a Vickers microhardness tester (HMV-G Series; Shimadzu Corp., Kyoto, Japan) with a pyramidal diamond indenter, using a load of 4.903 N and loading time of 5 s.

### 2.6. Statistical Analysis

Prior to the parametric analysis, the normality of the data distribution was verified using the Shapiro–Wilk test (*p* > 0.05), and the homogeneity of variances was assessed using Levene’s test (*p* = 0.64$). As both assumptions were satisfied, a three-way analysis of variance (ANOVA) was conducted to assess the statistical significance of the SBS results and to elucidate the effects of aging conditions, application of primer and cement systems, and their potential interactions. For the evaluation of the flexural strength and Vickers hardness, the normality of the data was confirmed (Shapiro–Wilk test, *p* > 0.05), and the homogeneity of variances was verified (Levene’s test, *p* = 0.506 for flexural strength; *p* = 0.457 for Vickers hardness). A two-way ANOVA was applied to determine the effects of the cement type and aging conditions. For the physical property tests (Flexural Strength and Vickers Hardness), post hoc comparisons were performed using Tukey’s test at a significance level of 0.05. All statistical analyses were performed using GraphPad Prism software (version 10.4.0; GraphPad Software Inc., San Diego, CA, USA).

## 3. Results

### 3.1. Shear Bond Strength

**Figure 2:**
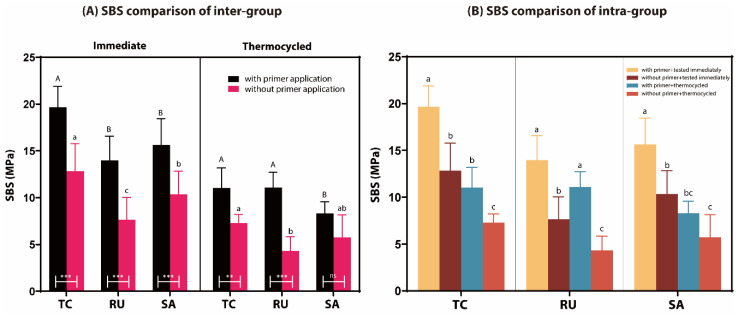
SBS test of experimental groups. (**A**) Inter-group comparison. Significant differences between experimental groups based on primer usage are denoted by uppercase letters, while those without primer application are indicated by lowercase letters within the same aging condition. *** *p* < 0.0001; ** *p* < 0.001; ns, no significant difference. (**B**) Intra-group comparison. Different lowercase letters mean significant difference within each experimental group.

The SBS test results for all the experimental groups are presented in Table 2 and Figure 2. The results indicated that the SBS between the zirconia surface and all the experimental resin cements decreased after thermocycling (*p* < 0.0001). In addition, the SBS significantly increased after the application of zirconia primers across all resin cements (*p* < 0.0001).

In the immediate testing group, all groups demonstrated a higher bond strength when primer was applied (*p* < 0.0001) (Figure 2A). In the groups primed using Z-Prime Plus, TC/P exhibited a significantly higher bond strength than RU/P and SA/P (*p* < 0.0001 and *p* = 0.0002, respectively), whereas RU/P and SA/P exhibited no significant difference in bond strength (*p* = 0.6352). Among the groups without primers, TC exhibited a significantly higher bond strength than RU and SA (*p* < 0.0001). The application of primer resulted in a significant increase in the SBS across all groups (*p* < 0.0001).

After thermocycling, both the TC/P and RU/P groups exhibited significantly higher SBS values than the SA/P group (*p* = 0.0387 and *p* = 0.0436, respectively), with no significant difference between the TC/P and RU/P groups. Furthermore, the TC group displayed a significantly greater SBS than the RU group (*p* = 0.0191), whereas no significant differences were detected between the RU and SA groups (*p* = 0.8289) or between the TC and SA groups (*p* = 0.7434). Primer application significantly increased SBS in the TC/P (*p* < 0.001) and RU/P (*p* < 0.0001) groups after thermocycling. However, there was no significant difference between the SA/P and SA groups (*p* = 0.0967).

Across all experimental groups, SBS values increased when a primer was applied compared with those under non-priming conditions (Figure 2B). Under primed conditions, the SBS of the TC/P and SA/P groups decreased after thermocycling (*p* < 0.0001), except for the RU group (*p* = 0.0502). Under non-primed conditions, SBS decreased across all experimental groups after thermocycling (*p* < 0.0126).

### 3.2. Failure Mode Analysis

**Figure 3:**
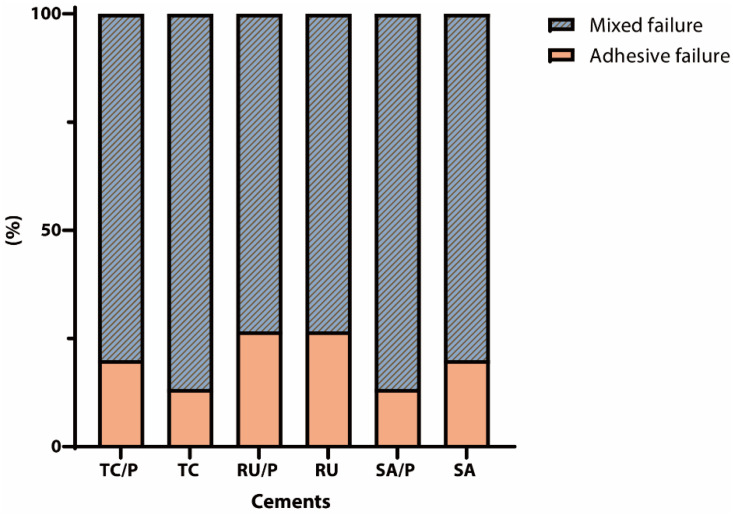
Percentages of failure modes in each group. Failures were classified as adhesive (failure at the zirconia–resin cement interface) or mixed (adhesive failure combined with cohesive failure involving >25% of the bonded surface). Groups represent the resin cement type (TheraCem (TC), Rely X U200 (RU), and Clearfil SA Luting (SA)) without and with primer application (TC/P, RU/P, and SA/P).

Figure 3 illustrates the distribution of failure modes among the specimens. As shown in Table 3, the premature failure rates in the RU and SA groups were 13.3% and 6.7%, respectively, whereas no premature failures were observed in the other groups. Failure mode analysis indicated that the majority of the specimens exhibited mixed failure with a low incidence of adhesive failure at the interface between the zirconia and resin cement. Cohesive failure was not observed in any experimental group. Adhesive failure occurred more frequently in the RU and RU/P groups than in other groups.

### 3.3. Fracture Interfacial Surface Analysis with FE-SEM

**Figure 4:**
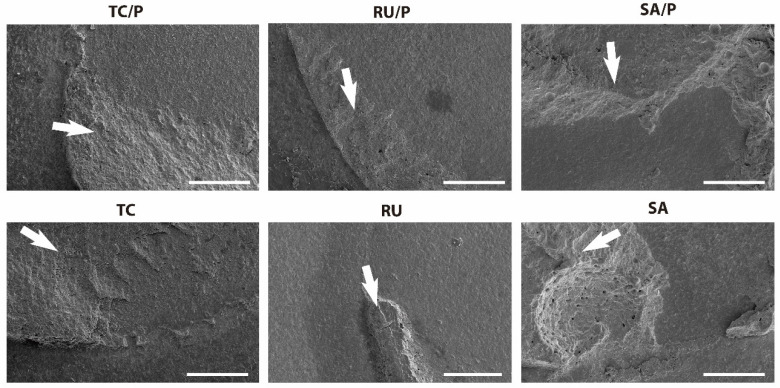
Representative SEM images (×500) of debonded zirconia surfaces following SBS tests for TC/P, TC, RU/P, RU, SA/P, and SA groups. TC/P and TC exhibited mixed failures with flame-shaped residual cement. RU/P and RU showed adhesive failures with smooth zirconia and localized resin remnants (arrows). SA/P and SA demonstrated mixed failures with stepped cohesive fractures and adhesive areas. Arrows indicate resin cement remnants; scale bar = 2 μm.

SEM images of the debonding surfaces of the TC/P, TC, RU/P, RU, SA/P, and SA specimens following the SBS test are presented in Figure 4. In the RU and RU/P groups, adhesive failure modes characterized by smooth zirconia surfaces were observed, with arrows indicating areas of residual resin cement. In contrast, the TC and TC/P groups exhibited mixed failure modes regardless of the zirconia primer application, with cohesive failures predominantly within the resin cement and a greater amount of residual material displaying a flame-shaped morphology. Similarly, the SA and SA/P groups demonstrated mixed failure modes, with cohesive failures evident in the residual resin cement and presenting a stepped fracture pattern along with adhesive failures on the smooth zirconia surfaces. In all images, the arrows highlight regions containing residual resin cement.

### 3.4. Flexural Strength Test

**Figure 5:**
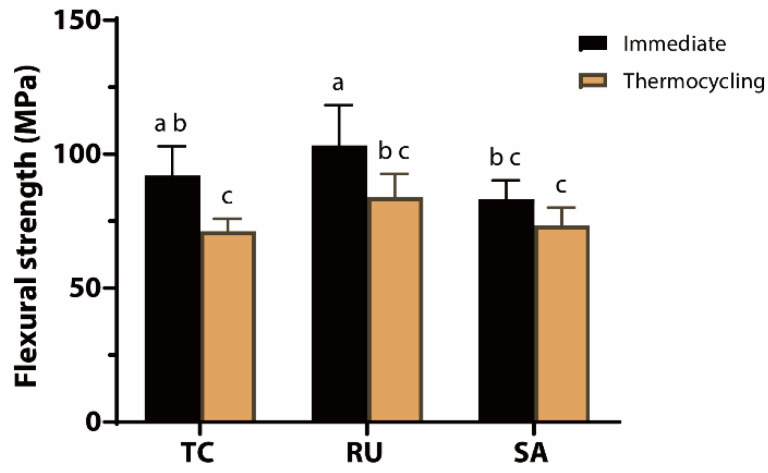
Flexural strength values for each cement under immediate and thermocycling conditions. Flexural strength values (MPa) are presented as mean ± standard deviation for TheraCem (TC), Rely X U200 (RU), and Clearfil SA Luting (SA). Specimens were tested either immediately after storage or after thermocycling. Different lowercase letters indicate statistically significant differences among groups (*p* < 0.05).

The mean flexural strengths for each cement type are shown in Figure 5. The flexural strength of all tested groups decreased significantly following thermocycling (*p* = 0.0002). Under the immediate testing condition, the RU group exhibited significantly higher flexural strength than the SA group (*p* = 0.0128), whereas no difference was observed when compared with the TC group (*p* = 0.2750). Under aging conditions, there were no differences in strength among the three experimental groups (*p* ≥ 0.1727).

### 3.5. Vickers Hardness Test

**Figure 6:**
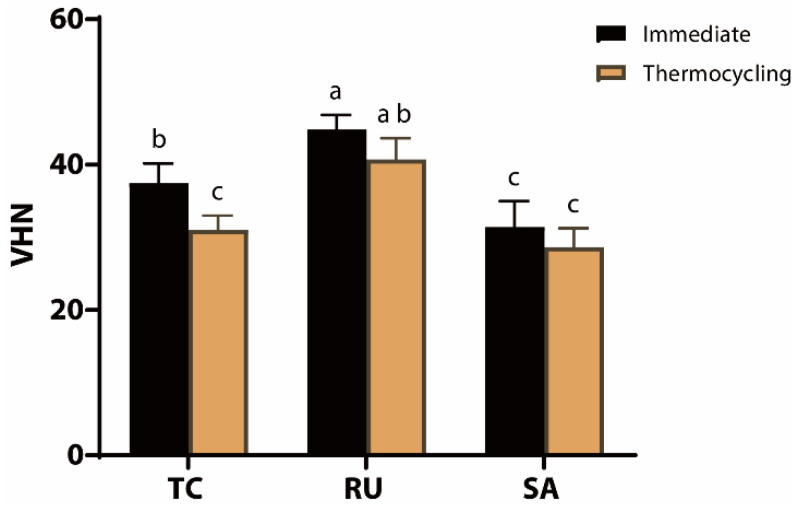
Vickers hardness value (VHN) for each cement under immediate and thermocycling conditions. Values are presented as mean ± standard deviation for TheraCem (TC), Rely X U200 (RU), and Clearfil SA Luting (SA). Specimens were tested either immediately after storage or after thermocycling. Different lowercase letters indicate statistically significant differences among groups (*p* < 0.05).

The mean Vickers hardness number (VHN) for each cement type is shown in Figure 6. After thermocycling, the TC group showed a statistically significant reduction in VHN (*p* = 0.0023), whereas the other groups did not exhibit a significant decrease (RU, *p* = 0.0649; SA, *p* = 0.3384). Under the immediate testing conditions, the RU group exhibited the highest hardness (44.825 VHN), whereas the lowest hardness was observed in the SA group (31.40 VHN). After thermocycling, the RU group maintained significantly higher hardness (40.713 VHN) than the TC and SA groups; however, the difference between the TC and SA groups was not statistically significant (*p* = 0.5064).

## 4. Discussion

Although reliable adhesion to zirconia remains challenging, the development of SARCs incorporating functional monomers such as 10-MDP aims to simplify clinical procedures while improving the initial bonding strength to zirconia. In this study, the material characteristics were evaluated with the aim of interpreting the observed bonding behavior and its changes following aging, rather than reiterating the general adhesion principles. Notably, the three-way ANOVA results (Table 2) demonstrated that cement type, primer application, and thermocycling each exerted a statistically significant effect on SBS (*p* < 0.0001), leading to the rejection of all corresponding null hypotheses.

In this study, SBS tests showed that the application of a zirconia primer led to a statistically significant increase in bond strength (*p* < 0.005). Under immediate testing conditions, the TC/P group had the highest SBS, whereas the RU group had the lowest. After thermocycling, both the TC/P and RU/P groups maintained relatively high bond strengths. Notably, the RU/P group showed only a 20% reduction in SBS after thermocycling, compared with a 40% reduction in the other groups (Figure 2). The SBS between zirconia and all types of SARCs increased significantly with the application of a zirconia primer, both initially and following thermocycling. Although no significant three-way interaction among cement type, primer application, and aging was observed, significant two-way interactions were identified for cement × aging and cement × primer (Table 2). These findings indicate that the effects of thermocycling and primer application are material-dependent rather than uniform across all SARCs. Overall, the bonding performance of SARCs to zirconia was primarily governed by material-specific two-way interactions rather than by complex three-factor interactions. This result supports previous studies showing that the application of primers can enhance the bond strength of zirconia restorations [20,25,42].

The enhancement of zirconia bonding afforded by MDP-containing primers can be attributed to well-defined chemical mechanisms. The phosphate moiety of 10-MDP interacts strongly with the zirconium oxide (ZrO_2_) layer on Y-TZP, forming stable Zr–O–P covalent linkages [43]. Evidence from spectroscopic investigations performed on bulk zirconia surfaces, including X-ray photoelectron spectroscopy (XPS) and solid-state nuclear magnetic resonance (NMR), supports the formation of these interfacial species and helps explain the improved chemical affinity and durability achieved with MDP-containing primers [9,44].

The SBS results demonstrated that thermocycling induced a significant reduction in bond strength across all SARC groups, regardless of primer application. Previous reports attribute such reductions in bond strength to the hydrophilic nature of SARCs, which promotes water uptake and hydrolytic degradation at the resin–zirconia interface, as well as to temperature-driven material changes [45]. Water diffusion at the bonding interface weakens adhesion via hydrolysis, and thermal stresses arising from the mismatch in the coefficients of thermal expansion between zirconia and resin cement further exacerbate interfacial degradation [46]. Notably, although the RU/P and TC/P groups exhibited comparable SBS values after thermocycling, both remained significantly lower than that of the TC group, consistent with previous evidence that MDP-related primer interactions and MDP-salt formation can influence the durability of zirconia bonding under aging conditions [47,48]. The acidic phosphate monomers in RU are inherently hydrophilic, which promotes water sorption and diffusion into the bonded interface, thereby accelerating hydrolytic degradation and compromising the long-term stability of the resin–zirconia bond. The inherent hydrophilicity of the acidic phosphate monomers renders their bonding interfaces susceptible to water ingress and hydrolytic degradation, particularly under thermal stress. However, the application of an MDP-containing primer appeared to counteract this weakness by establishing stable chemical complexes with the zirconia surface, thereby delaying interfacial water diffusion. Consequently, although all groups exhibited significant SBS reductions after thermocycling, the RU/P specimens demonstrated the smallest decline (approximately 20.7%), indicating superior maintenance of the bond integrity. This highlights the clinical value of MDP-based primers in enhancing the longevity of SARCs under variable thermal conditions in the intraoral environment.

Failure-mode analysis (Figure 3) demonstrated that adhesive debonding was most frequent in the RU and RU/P groups, each exhibiting 26.7% adhesive failures. Notably, the RU group subjected to thermocycling exhibited a particularly high rate of premature failure (Table 3), underscoring its inadequate resistance to cyclic thermal stress at the resin–zirconia interface. The FE-SEM images corroborate this observation, revealing pronounced interfacial separation and minimal remnants of cohesive resin on the zirconia surface. Conversely, mixed failure modes predominated across all cement formulations regardless of primer application, indicating a combination of cohesive and adhesive failure mechanisms. The premature failures observed in the RU and SA groups could be attributed to their relatively low bond strengths, which were insufficient to endure the thermodynamic challenges imposed by thermocycling. The premature failure rates in this study highlight the vulnerability of weak adhesive interfaces, particularly after thermocycling. These failures were included in the SBS analysis as zero strength values to avoid overestimation and to provide a conservative, clinically relevant assessment of interfacial durability [49,50].

The results of the flexural and Vickers hardness tests can be explained by the fact that RU contains more inorganic fillers (43 vol%, 72 wt%) than TC (about 40 vol%, 60–65 wt%) and SA (44 vol%, 65 wt%) [51]. After thermocycling, the flexural strength and Vickers hardness of the SARCs decreased owing to water absorption and matrix swelling. The results of this study are consistent with those of previous studies [38,40], indicating that assessments of physical properties should be conducted along with bond strength evaluations. The Vickers hardness is an important property that can affect clinical outcomes. Materials with a lower surface hardness may deteriorate more easily, causing fatigue of the material and decreasing its clinical survival. The RU group, characterized by the highest inorganic filler loading, had a significantly higher flexural strength and Vickers hardness than the SA group, which was consistent with its SBS performance. Despite its lower filler content relative to that of RU, the TC group achieved higher SBS values, although its flexural strength and hardness were significantly inferior to those of the RU group. This apparent divergence between mechanical properties and adhesive performance can be explained by the distinct chemistry of the TC formulation. Unlike other SARCs, TC incorporates calcium silicate, which is an alkaline mineral phase that buffers acidification at the bonding interface. Acidic microenvironments, often arising from resin polymerization or oral acidic challenges, enhance water sorption and hydrolytic degradation, thereby undermining the bond integrity [52]. The alkaline milieu provided by calcium silicate counteracts this effect by reducing water uptake and stabilizing the interface. Moreover, functional monomers, such as MDP, exhibit enhanced chelation and chemical activity under neutral-to-alkaline conditions, further strengthening the resin–zirconia bond [34,53]. As a result, TC not only maintained superior SBS across all aging conditions but also exhibited a statistically significant increase in SBS when used in conjunction with an MDP-containing primer. These findings highlight the dual importance of the filler composition and primer chemistry in optimizing the durability of SARCs under intraoral thermal and hydrolytic stresses. Clinically, the incorporation of alkaline fillers with MDP primers may offer a robust strategy for improving the long-term performance of zirconia-based restorations.

In response to these ongoing discussions, a newer subgroup of SARCs has been introduced that combines functional phosphate monomers with calcium-silicate-related components and exhibits an early acid-to-alkaline shift during setting. Notably, while MDP-containing resin cements are already widely available (e.g., Panavia F 2.0, Panavia V5, Rely X Unicem 2, SpeedCEM Plus, and G-CEM One), TC has been highlighted as a calcium-silicate-based self-adhesive cement showing alkaline behavior after polymerization [34]. This alkaline shift is clinically relevant because it may promote a more favorable chemical environment for phosphate-mediated interactions at oxide ceramic surfaces and improve resistance to water-related degradation compared with conventional SARCs that rely on phosphate chemistry alone. However, durability remains material- and protocol-dependent and requires aging validation [54]. SARCs containing calcium silicate continuously release fluoride and calcium ions, contributing to a rapid shift in the surrounding environment from acidic to alkaline [55]. The resulting alkalinity played a crucial role in neutralizing hydrogen ions, thereby creating favorable conditions for chemical interactions. Specifically, an alkaline environment enhances the coordination between MDP and the zirconia surface, ultimately promoting improved chemical bonding and interfacial stability [53].

In addition to the alkaline characteristics of TC, the sustained release of calcium ions may contribute to the longevity of cemented zirconia restorations. Chen et al. [34] reported that TC continuously releases calcium ions for up to 56 days. This prolonged calcium-ion release may facilitate the formation of more stable MDP–calcium complexes at the interface between the cement and primed zirconia surface, thereby indirectly enhancing the interfacial stability rather than promoting chemical bonding to zirconia. Based on these findings, manufacturers have suggested that SARCs incorporating both MDP and calcium silicate can be applied effectively without the use of primer [25].

Although the application of an MDP-containing primer improved the initial bond strength to zirconia in the present study, thermocycling still produced a significant reduction in SBS. This decline is most likely attributed to the hydrolytic instability of the Zr–O–P bonds [24,56,57] and the lack of acid neutralization at the resin–zirconia interface [14,58]. The chemical interactions between the dihydrogen phosphate group of 10-MDP and zirconium oxide are based on the Zr–O–P coordinate bond, which is inherently susceptible to hydrolysis in aqueous environments. This interaction undergoes time-dependent degradation, as evidenced by phosphorus release during water storage, and thermodynamic analyses have shown a tendency toward bond dissociation under neutral or acidic conditions [24,56]. Moreover, unlike hydroxyapatite, zirconia lacks a buffering capacity and cannot neutralize acidic MDP monomers at the interface. While MDP reacts with calcium-containing substrates to form stable water-insoluble MDP–calcium salts, the chemically inert zirconia surface maintains a persistently acidic interphase, which may compromise resin polymerization and accelerate hydrolytic degradation [14]. In addition, water ingress and competitive adsorption at hydroxyl sites on the zirconia surface can progressively displace phosphate groups, thereby undermining the interfacial stability over time [56,57]. Collectively, these findings indicate that although 10-MDP is considered the gold-standard functional monomer for zirconia bonding, MDP-mediated adhesion remains vulnerable to hydrolytic degradation, which explains the significant decrease in bond strength observed in this study after thermocycling despite primer application.

In this study, the frequent occurrence of mixed failures indicates that cohesive failure within the resin cement contributed substantially to debonding, suggesting that the zirconia–resin cement interface was not a weak link. This interpretation is consistent with prior research showing that MDP-containing primers can strengthen zirconia bonding and shift failure modes toward mixed/cohesive failure modes, reflecting improved interfacial integrity [25]. After thermocycling, the concurrent reduction in the SBS and mechanical properties can be explained by the hydrothermal degradation of the resin cement matrix, which increases the susceptibility to cohesive failure [58]. Thus, in addition to enhancing the initial chemical bonding to zirconia, the application of an MDP-containing primer may increase the likelihood of failure occurring within the cement rather than purely at the interface. However, thermocycling-driven cement degradation can still reduce the overall bond durability despite the improvement in the initial interfacial bond strength.

This study has several limitations. First, all experiments were conducted in vitro, and the simplified aging protocol may not fully capture the complex mechanical, chemical, and thermal stresses present in the oral environment. Second, this study evaluated only three commercially available SARCs and a single zirconia formulation, thereby limiting the generalizability of the results to other cement systems and surface treatments. Finally, the bond strength was assessed only immediately and following artificial aging via thermocycling. Despite these limitations, the present study clearly demonstrated that primer application significantly enhanced bonding performance. Moreover, the relatively new MDP-containing SARC formulation, such as TC, exhibited superior bond strength compared with conventional acidic-based SARCs. Nonetheless, further long-term and in vivo studies are warranted to validate the clinical durability of MDP-containing SARCs and the associated primer protocols. In addition, future studies should investigate the bonding performance at the cement–tooth interface, including to enamel and dentin, to better reflect clinical conditions. Evaluating a broader range of SARCs, such as other MDP-containing systems, would also help to clarify the generalizability of the present findings.

## 5. Conclusions

A reduction in bond strength after artificial aging was observed in all groups, indicating that aging-related degradation could not be fully prevented. Within the limitations of this study, MDP-containing SARCs demonstrated superior bonding performance compared with non-MDP formulations. The application of a zirconia primer enhanced the bond strength of all tested SARCs, highlighting the importance of chemical interactions at the zirconia–cement interface. Nevertheless, a reduction in bond strength after thermocycling was observed in all groups, indicating that aging-related degradation could not be fully prevented.

## Figures and Tables

**Table 1:** Compositions of the materials used in the study.

Material	Main Composition	Manufacturer	Lot No.
MazicZir (Zirconia disc)	97% Zirconium dioxide stabilized with 3% Y-TZP ceramics	Vericom, Chuncheon, Republic of Korea	DMS220146J50
Ethanol	99% Ethanol	Jin Chemical Co., Ltd., Siheung, Republic of Korea	A380807
Z-prime plus	MDP, bis-GMA, 2-hydroxyethyl methacrylate, ethanol, triethylamine	BISCO Inc., Schaumburg, IL, USA	2300111953
Rely X U200 (RU)	Glass powder, 2-propenoic acid, 2-methyl-, 1,1′[1-(hydroxymethyl)-1,2ethanediyl] ester, reaction products with 2-hydroxy-1,3propanediyl dimethacrylate and phosphorus oxide, 2,2′-ethylenedioxydiethyl dimethacrylate, silane treated silica, sodium persulfate, oxide glass, tert-butyl 3,5,5-trimethylperoxyhexanoate, acetic acid, copper salt, monohydrate	3M ESPE, Neuss, Germany	10503980
TheraCem (TC)	Catalyst: 10-MDP, 2-hydroxyethyl methacrylate, tert-butyl perbenzoate Base: Portland cement, ytterbium with barium glass, ytterbium fluoride, bis-GMA	BISCO Inc., Schaumburg, IL, USA	2300111554
Clearfil SA Luting (SA)	Bis-GMA, TEGDMA, MDP, hydrophobic armomatic dimethacrylate, silanated barium glass filler, silanated colloidal silica, di-camphorquinone, benzoyl peroxide, initiator, accelerators	Kuraray Noritake Dental Inc., Okayama, Japan	B10266

Y-TZP, Yttria-stabilized tetragonal zirconia polycrystal; MDP, 10-methacryloyloxydecyl dihydrogen phosphate; Bis-GMA, bisphenol A-glycidyl methacrylate; TEGDMA, triethylene glycol dimethacrylate; SA, self-adhesive (similar to the self-adhesive resin cement).

**Table 2:** Three-way ANOVA test results.

ANOVA Table	*F* Value	*p* Value
Cement	39.40	<0.0001
Aging	293.2	<0.0001
Primer	336.1	<0.0001
Cement × Aging	14.14	<0.0001
Cement × Primer	6.900	0.0026
Aging × Primer	5.863	0.0199
Cement × Aging × Primer	2.292	0.1136

Three-way ANOVA results showing *F*-values and corresponding significance levels (*p*-values) for cement type, aging, use of primer, and their interactions.

**Table 3:** Premature failure rates in each experimental group.

	RU	TC	SA
With primer application	0	0	0
Without primer application	2 (13.3%)	0	1 (6.7%)

## Data Availability

The data presented in this study are available upon request from the corresponding author.

## References

[1] Ling L., Ma Y., Chen Y., Malyala R. (2022). Physical, Mechanical, and Adhesive Properties of Novel Self-Adhesive Resin Cement. Int. J. Dent..

[2] Saravia-Rojas M.Á., Nima G., Geng-Vivanco R., Abuna G.F., Tay L.Y., Puppin-Rontani R.M. (2021). Limited Etching Time Increases Self-Adhesive Resin Cement Adhesion to Enamel. Oper. Dent..

[3] Heboyan A., Vardanyan A., Karobari M.I., Marya A., Avagyan T., Tebyaniyan H., Mustafa M., Rokaya D., Avetisyan A. (2023). Dental Luting Cements: An Updated Comprehensive Review. Molecules.

[4] Hosaka K., Tagami J., Nishitani Y., Yoshiyama M., Carrilho M., Tay F.R., Agee K.A., Pashley D.H. (2007). Effect of Wet vs. Dry Testing on the Mechanical Properties of Hydrophilic Self-Etching Primer Polymers. Eur. J. Oral Sci..

[5] Alqutaibi A.Y., Ghulam O., Krsoum M., Binmahmoud S., Taher H., Elmalky W., Zafar M.S. (2022). Revolution of Current Dental Zirconia: A Comprehensive Review. Molecules.

[6] Carpena Lopes G., Spohr A.M., De Souza G.M. (2016). Different Strategies to Bond Bis-GMA–Based Resin Cement to Zirconia. J. Adhes. Dent..

[7] Kern M., Wegner S.M. (1998). Bonding to Zirconia Ceramic: Adhesion Methods and Their Durability. Dent. Mater..

[8] Thompson J.Y., Stoner B.R., Piascik J.R., Smith R. (2011). Adhesion/Cementation to Zirconia and Other Non-Silicate Ceramics: Where Are We Now?. Dent. Mater..

[9] Nagaoka N., Yoshihara K., Feitosa V.P., Tamada Y., Irie M., Yoshida Y., Van Meerbeek B., Hayakawa S. (2017). Chemical Interaction Mechanism of 10-MDP with Zirconia. Sci. Rep..

[10] Shen D., Wang H., Shi Y., Su Z., Hannig M., Fu B. (2023). The Effect of Surface Treatments on Zirconia Bond Strength and Durability. J. Funct. Biomater..

[11] Yoshihara K., Nagaoka N., Hayakawa S., Okihara T., Yoshida Y., Van Meerbeek B. (2018). Chemical Interaction of Glycero-Phosphate Dimethacrylate (GPDM) with Hydroxyapatite and Dentin. Dent. Mater..

[12] Yoshihara K., Hayakawa S., Nagaoka N., Okihara T., Yoshida Y., Van Meerbeek B. (2018). Etching Efficacy of Self-Etching Functional Monomers. J. Dent. Res..

[13] Van Meerbeek B., Yoshihara K., Van Landuyt K., Yoshida Y., Peumans M. (2020). From Buonocore’s Pioneering Acid-Etch Technique to Self-Adhering Restoratives: A Status Perspective of Rapidly Advancing Dental Adhesive Technology. J. Adhes. Dent..

[14] Carrilho E., Cardoso M., Marques Ferreira M., Marto C.M., Paula A., Coelho A.S. (2019). 10-MDP-Based Dental Adhesives: Adhesive Interface Characterization and Adhesive Stability—A Systematic Review. Materials.

[15] Spori D.M., Venkataraman N.V., Tosatti S.G., Durmaz F., Spencer N.D., Zürcher S. (2007). Influence of Alkyl Chain Length on Phosphate Self-Assembled Monolayers. Langmuir.

[16] Van Landuyt K.L., Peumans M., De Munck J., Lambrechts P., Van Meerbeek B. (2006). Extension of a One-Step Self-Etch Adhesive into a Multi-Step Adhesive. Dent. Mater..

[17] Manso A.P., Carvalho R.M. (2017). Dental Cements for Luting and Bonding Restorations: Self-Adhesive Resin Cements. Dent. Clin. N. Am..

[18] Lee S.E., Bae J.H., Choi J.W., Jeon Y.C., Jeong C.M., Yoon M.J., Huh J.B. (2015). Comparative Shear-Bond Strength of Six Dental Self-Adhesive Resin Cements to Zirconia. Materials.

[19] de Souza G., Hennig D., Aggarwal A., Tam L.E. (2014). The Use of MDP-Based Materials for Bonding to Zirconia. J. Prosthet. Dent..

[20] Yi Y.-A., Ahn J.-S., Park Y.-J., Jun S.-H., Lee I.-B., Cho B.-H., Son H.-H., Seo D.-G. (2015). The Effect of Sandblasting and Different Primers on Shear Bond Strength between Yttria-Tetragonal Zirconia Polycrystal Ceramic and a Self-Adhesive Resin Cement. Oper. Dent..

[21] Steiner R., Heiss-Kisielewsky I., Schwarz V., Schnabl D., Dumfahrt H., Laimer J., Steinmassl O., Steinmassl P.-A. (2020). Zirconia Primers Improve the Shear Bond Strength of Dental Zirconia. J. Prosthodont..

[22] Madruga F.C., Ogliari F.A., Ramos T.S., Bueno M., Moraes R.R. (2013). Calcium hydroxide, pH-neutralization and formulation of model self-adhesive resin cements. Dent. Mater..

[23] Roedel L., Zorzin J., Petschelt A., Lohbauer U. (2017). Hygroscopic Expansion Stress and pH Neutralization of Self-Adhesive Resin Cements. Clin. Oral Investig..

[24] Yue X., Hou X., Gao J., Bao P., Shen J. (2019). Effects of MDP-Based Primers on Shear Bond Strength between Resin Cement and Zirconia. Exp. Ther. Med..

[25] Lim M.-J., Yu M.-K., Lee K.-W. (2018). The Effect of Continuous Application of MDP-Containing Primer and Luting Resin Cement on Bond Strength to Tribochemical Silica-Coated Y-TZP. Restor. Dent. Endod..

[26] Nakayama D., Koizumi H., Komine F., Blatz M.B., Tanoue N., Matsumura H. (2010). Adhesive Bonding of Zirconia with Single-Liquid Acidic Primers and a Tri-n-Butylborane Initiated Acrylic Resin. J. Adhes. Dent..

[27] Koizumi H., Nakayama D., Komine F., Blatz M.B., Matsumura H. (2012). Bonding of Resin-Based Luting Cements to Zirconia with and without the Use of Ceramic Priming Agents. J. Adhes. Dent..

[28] Shin Y.-J., Shin Y., Yi Y.-A., Kim J., Lee I.-B., Cho B.-H., Son H.-H., Seo D.-G. (2014). Evaluation of the Shear Bond Strength of Resin Cement to Y-TZP Ceramic after Different Surface Treatments. Scanning.

[29] Ahn J.-S., Yi Y.-A., Lee Y., Seo D.-G. (2015). Shear Bond Strength of MDP-Containing Self-Adhesive Resin Cement and Y-TZP Ceramics: Effect of Phosphate Monomer-Containing Primers. BioMed Res. Int..

[30] Afrasiabi A., Mostajir E., Golbari N. (2018). The Effect of Z-Prime on the Shear Bond Strength of Zirconia Ceramic to Dentin: In Vitro. J. Clin. Exp. Dent..

[31] Go E., Shin Y., Park J. (2019). Evaluation of the Microshear Bond Strength of MDP-Containing and Non–MDP-Containing Self-Adhesive Resin Cement on Zirconia Restoration. Oper. Dent..

[32] Kuraray Noritake Dental Inc. (2013). CLEARFIL™ SA LUTING Technical Guide.

[33] Saskalauskaite E., Tam L.E., McComb D. (2008). Flexural Strength, Elastic Modulus, and pH Profile of Self-Etch Resin Luting Cements. J. Prosthodont..

[34] Chen L., Yang J., Wang J.R., Suh B.I. (2018). Physical and Biological Properties of a Newly Developed Calcium Silicate-Based Self-Adhesive Cement. Am. J. Dent..

[35] Naveen K.S., Singh J.P., Viswambaran M., Dhiman R.K. (2015). Evaluation of Flexural Strength of Resin Interim Restorations Impregnated with Various Types of Silane-Treated and Untreated Glass Fibres. Med. J. Armed Forces India.

[36] Nakamura T., Wakabayashi K., Kinuta S., Nishida H., Miyamae M., Yatani H. (2010). Mechanical Properties of New Self-Adhesive Resin-Based Cement. J. Prosthodont. Res..

[37] Li J. (2011). Effect of Flexural Strength of Orthodontic Resin Cement on Bond Strength of Metal Brackets to Enamel Surfaces. Eur. J. Orthod..

[38] Poggio C., Lombardini M., Gaviati S., Chiesa M. (2012). Evaluation of Vickers Hardness and Depth of Cure of Six Composite Resins Photo-Activated with Different Polymerization Modes. J. Conserv. Dent..

[39] Fischer H., Marx R. (2002). Fracture Toughness of Dental Ceramics: Comparison of Bending and Indentation Method. Dent. Mater..

[40] Ozdogan A., Yesil Duymus Z. (2020). Investigating the Effect of Different Surface Treatments on Vickers Hardness and Flexural Strength of Zirconium and Lithium Disilicate Ceramics. J. Prosthodont..

[41] (2019). Dentistry—Polymer-Based Restorative Materials.

[42] Valente F., Mavriqi L., Traini T. (2020). Effects of 10-MDP-Based Primer on Shear Bond Strength between Zirconia and New Experimental Resin Cement. Materials.

[43] Suh W.K., Kim K.-M., Kwon J.-S. (2023). Changes in Adhesive Strength and pH of Dental Universal Adhesive in Accordance with Varying Proportions of 10-MDP. Korean J. Dent. Mater..

[44] Chen Y., Lu Z., Qian M., Zhang H., Xie H., Chen C. (2017). Effect of 10-Methacryloxydecyl Dihydrogen Phosphate Concentration on Chemical Coupling of Methacrylate Resin to Yttria-Stabilized Zirconia. J. Adhes. Dent..

[45] da Silva E.M., Miragaya L., Sabrosa C.E., Maia L.C. (2014). Stability of the Bond between Two Resin Cements and an Yttria-Stabilized Zirconia Ceramic after Six Months of Aging in Water. J. Prosthet. Dent..

[46] Wegner S.M., Gerdes W., Kern M. (2002). Effect of Different Artificial Aging Conditions on Ceramic–Composite Bond Strength. Int. J. Prosthodont..

[47] Tsuda F., Yoshida K., Sawase T. (2024). Effects of Primer Components of Silane and 10-Methacryloyloxydecyl Dihydrogen Phosphate on Resin Bonding to Tribochemical Silica-Coated Highly Translucent Zirconia. Clin. Oral Investig..

[48] Abdou A., Hussein N., Abd El-Sattar N.E.A., Takagaki T., Kusumasari C., Rizk A., Abo-Alazm E.A. (2023). MDP-Salts as an Adhesion Promoter with MDP-Primers and Self-Adhesive Resin Cement for Zirconia Cementation. BMC Oral Health.

[49] Franz A., Lettner S., Watts D.C., Graf A., Moritz A., Schedle A. (2018). Analysis of Pre-Test Failures and Bond-Strengths of Seven Adhesive Systems to Bovine Dentine: A Nine-Year Novice/Beginner Operator Study. Dent. Mater..

[50] Bitter K., Neumann K., Kielbassa A.M. (2008). Effects of Pretreatment and Thermocycling on Bond Strength of Resin Core Materials to Various Fiber-Reinforced Composite Posts. J. Adhes. Dent..

[51] Berkman M., Tuncer S., Tekce N., Karabay F., Demirci M. (2021). Microtensile Bond Strength between Self-Adhesive Resin Cements and Resin-Based Ceramic CAD/CAM Block. Odovtos-Int. J. Dent. Sci..

[52] Zorzin J., Petschelt A., Ebert J., Lohbauer U. (2012). pH Neutralization and Influence on Mechanical Strength in Self-Adhesive Resin Luting Agents. Dent. Mater..

[53] Xie H., Tay F.R., Zhang F., Lu Y., Shen S., Chen C. (2015). Coupling of 10-Methacryloyloxydecyldihydrogenphosphate to Tetragonal Zirconia: Effect of pH Reaction Conditions on Coordinate Bonding. Dent. Mater..

[54] Yang L., Chen B., Xie H., Chen Y., Chen Y., Chen C. (2018). Durability of Resin Bonding to Zirconia Using Products Containing 10-Methacryloyloxydecyl Dihydrogen Phosphate. J. Adhes. Dent..

[55] Chen L., Gleave C., Suh B. New Self-Adhesive Resin Cement with Alkaline pH. Proceedings of the 2017 IADR/AADR/CADR General Session.

[56] Chen C., Chen Y., Lu Z., Qian M., Xie H., Tay F.R. (2017). The Effects of Water on Degradation of the Zirconia–Resin Bond. J. Dent..

[57] Lee T.-H., Ahn J.-S., Shim J.-S., Han C.-H., Kim S.-J. (2011). Influence of Cement Thickness on Resin–Zirconia Microtensile Bond Strength. J. Adv. Prosthodont..

[58] Bürgin S., Rohr N., Fischer J. (2017). Assessing Degradation of Composite Resin Cements during Artificial Aging by Martens Hardness. Head Face Med..

